# The Association Between Longer Maternal Leukocyte Telomere Length in the Immediate Postpartum Period and Preterm Birth in a Predominately Latina Cohort of Mothers

**DOI:** 10.1007/s10995-025-04056-z

**Published:** 2025-02-19

**Authors:** Usah Dutson, Jue Lin, Laura L. Jelliffe-Pawlowski, Kimberly Coleman-Phox, Larry Rand, Janet M. Wojcicki

**Affiliations:** 1 Division of Pediatric Gastroenterology, Hepatology, and Nutrition, Department of Pediatrics, University of California San Francisco, UCSF Benioff Children’s Hospital, 550 16th Street, 5th Floor, Mail Code 0136, San Francisco, CA 94158 USA; 2 School of Public Health, University of California Berkeley, Berkeley, CA USA; 3 Department of Biochemistry and Biophysics, University of California, San Francisco, CA USA; 4 Department of Epidemiology and Biostatistics, University of California San Francisco, San Francisco, CA USA; 5 California Preterm Birth Initiative, University of California San Francisco, San Francisco, CA USA; 6 Department of Obstetrics, Gynecology and Reproductive Sciences, University of California, San Francisco, San Francisco, CA USA

**Keywords:** Maternal telomere length, Leukocyte telomere length, Preterm, Latina, Latinx, Hispanic

## Abstract

**Objectives:**

We investigated the association between maternal leukocyte telomere length (LTL) in the immediate postpartum period and moderate to late preterm birth (32– < 37 weeks) among Latinas, a population at high risk for preterm birth.

**Methods:**

Maternal LTL was measured using quantitative polymerase chain reaction at delivery in a prospective San Francisco primarily Latina birth cohort. Logistic regression models were used to investigate the association between postpartum maternal LTL and preterm birth. Maternal LTL was analyzed as a continuous predictor.

**Results:**

Out of 194 participants, 23 (11.9%) had preterm delivery. Longer postnatal maternal LTL was associated with preterm birth (crude OR 4.68; 95% confidence interval (CI) 1.07, 20.6, p = 0.039; adjusted OR 12.8, 95% CI 1.83, 99.9, p = 0.010). Age-stratified analysis showed that being under 35 years increased the effect size of the association between maternal LTL and preterm birth (adjusted OR 32.5, 95% CI 2.58, 597, p < 0.01).

**Conclusions for Practice:**

Latina mothers with moderate to late preterm infants had longer LTL in the immediate postpartum period compared to those with term infants. This association was stronger for mothers under the age of 35 years. LTL may serve as a biomarker to better understand the pathophysiology and risk of preterm birth and could inform targeted interventions for prevention and early detection. Future studies are needed to understand physiological changes in maternal LTL from the prenatal to postnatal period in relation to birth outcomes.

## Significance

Preterm birth is a significant global health issue, with certain populations, such as Latinas, at higher risk. While maternal telomere length (LTL) has been linked to preterm birth in other studies, our research uniquely investigates this association in a primarily Latina cohort. We found that longer postpartum maternal LTL was significantly associated with increased preterm birth risk, particularly in women under 35 years old. These findings contribute to the growing body of evidence on telomere dynamics in the perinatal period and suggest LTL may serve as a biomarker to better understand the pathophysiology of preterm birth and identify modifiable risk factors.

## Introduction

Preterm birth is a significant global public health concern, with over 15 million births occurring annually worldwide (Cao et al., 2022). The complications associated with preterm birth are a primary cause of mortality in children under the age of 5, resulting in approximately 1 million deaths (Perin et al., 2022). The etiology of preterm birth is multifactorial, involving a complex interplay of genetic, environmental, and socioeconomic factors (Cobo et al., 2020; Leonard et al., 2015). Latina women with gestational diabetes have a higher rate of preterm birth compared to the overall rate across all racial and ethnic groups (119.7 vs. 112.5 per 1000 live births). Their risk of preterm birth is also approximately 18% higher compared to white individuals (Venkatesh et al., 2022). Known risk factors for preterm birth include maternal age over 35 years, elevated body mass index (BMI), maternal smoking, maternal stress, poverty, low education and obstetrical factors (e.g., prior preterm birth, uterine curettage, cervical surgery, uterine malformations, short cervical length) (Cobo et al., 2020).

Recent studies have explored the association between telomere dynamics and preterm birth (Farladansky-Gershnabel et al., 2023; Marrs et al., 2018; Page et al., 2021; Schneper et al., 2023). Telomeres, a structure of repetitive DNA sequences at the ends of chromosomes, serve a crucial role in preserving the integrity and stability of the genome (Jafri et al., 2016). They prevent chromosomal degradation and DNA damage. Telomere shortening occurs gradually during cell division, leading to cellular senescence or apoptosis. Telomere length dynamics are regulated by several factors including oxidative stress and telomerase activity. Human telomerase reverse transcriptase (hTERT), which is a subunit of telomerase, adds ‘TTAGGG’ repeats to the ends of chromosomes to maintain telomere length (Jafri et al., 2016). Telomere attrition has been implicated in various age-related diseases including cardiovascular disease (Haycock et al., 2014). Conversely, an increase in telomere length due to inherited genetic variations is associated with an increased risk of developing site-specific cancers (Haycock et al., 2017).

Studies have shown an association between shorter maternal telomere length and preterm birth in other population groups (Farladansky-Gershnabel et al., 2023; Page et al., 2021; Panelli et al., 2022a). A study of 100 Mexican-origin participants found a significant association between shorter prenatal maternal leukocyte telomere length (LTL) and preterm birth (Page et al., 2021). Furthermore, maternal genetic variations in hTERT, a subunit of telomerase, have been identified as potential contributors to the risk of preterm birth (Marrs et al., 2018). Other studies have reported associations between shorter LTL and chronic disease associated with preterm birth including obesity and hypertension among the Latinx population (Incollingo Rodriguez et al., 2022; Kjaer et al., 2018; Wojcicki et al., 2018).

Our study examined the association between maternal LTL measured in the early postpartum period (at delivery) and preterm birth, exploring the relationship between LTL and preterm birth in a primarily Latinx birth cohort. Previous studies have investigated LTL in the prenatal period in relation to preterm birth (Page et al., 2021); however, LTL at delivery may be a better indicator of the combined impact of inflammation, and cellular aging, which are implicated in preterm birth pathogenesis. Assessing maternal postnatal LTL at the time of delivery may offer additional insight into risk for preterm birth.

## Methods

### Study Design and Participants

Data were used from an on-going, prospective birth cohort of primarily Latina mothers and their infants recruited from August 2018 to December 2022 to assess risk factors for preterm birth and childhood obesity. The study was conducted at two teaching hospitals in San Francisco including UCSF Benioff and Zuckerberg San Francisco General Hospitals. This study was approved by the Institutional Review Board (IRB) at the University of California, San Francisco (UCSF). All participants provided written informed consent for their participation and their children’s participations in the study. The study had been conducted in accordance with the ethical standards set forth in the 1964 Declaration of Helsinki and its subsequent amendments.

Participants were recruited during the postpartum period at both hospitals between 6 and 12 h postpartum and were explained that this was a study to assess the relationship between maternal LTL and neonatal and postnatal outcomes including preterm birth and infant growth trajectories. Initially our study included only the mothers who self-identified as Latina or had a partner that identified as Latino. However, after two months of recruitment, we decided to include all races and ethnicities to speed up our enrollment, but continued to oversample Latinx families as possible.

### Inclusion and Exclusion Criteria and Procedures

Mothers with moderate to late preterm (32 to 37 weeks) and term infants were included in the study (WHO). Mothers and infants were excluded if infants were born at < 32 weeks gestation, due to significantly higher morbidity and mortality rates (Glass et al., 2015) in this population group as another primary outcome of the study was to assess predictors (including LTL) of weight gain in the first year of life. We also excluded infants who were scheduled for immediate surgeries or had any contraindications for breastfeeding again related to our growth outcome of interest. Sample size calculation was based on infant growth outcomes at 6 months of age.

In the immediate postpartum period, a blood sample was obtained via fingerstick and placed on Whatman dried blood spot cards for the measurement of maternal LTL. LTL analysis was subsequently assessed by the Blackburn lab at UCSF using quantitative polymerase chain reaction (qPCR) as described below. During the postpartum period, mothers were also interviewed about dietary intake during pregnancy, socio-demographics, and maternal health history. Infant and maternal medical record were assessed to extract infant birth anthropometrics, delivery specifics (Cesarean versus vaginal delivery), gestational age and maternal medical diagnoses during pregnancy. In particular, maternal characteristics that were retrieved from medical records and/or surveyed via questionnaire included maternal age at the time of delivery, maternal and paternal self-reported Latinx ethnicity, maternal comorbidities (pre-existing diabetes mellitus and/or gestational diabetes mellitus, hypertension, depression and/or anxiety during pregnancy or pre-existing, and pre-pregnancy overweight or obesity), smoking status (being a smoker and/or exposed to secondhand smoke), marital status, education attainment, and consumption of sugar sweetened beverages (defined as fruit juices having less than 100% fruit, sodas, sweetened teas and fruit drinks) and 100% fruit juices. Birthweight and infant gestational age were extracted from the medical record. Weight-for-age categories, small for gestational age (SGA), appropriate for gestational age (AGA) or large for gestational age (LGA) for term infants were calculated using sex-specific percentiles from the WHO Growth Charts data (Centers for Disease Control and Prevention, n.d.), while the same categories for preterm infants were based on the Fenton Preterm Growth Chart 2013 (Fenton & Kim, 2013).

### Leukocyte Telomere Length Analysis

DNA was extracted from dried blood spots (DBS) on Whatmann 903 cards using the QIAamp DNA Investigator kit (QIAGEN cat# 56504) and eluted in 50 ul ATE buffer. The DBS were collected in three batches: from August 2018 to Nov 2020, from December 2021 to February 2022, and from June 2022 to November 2022. DBS were stored at – 80 °C and extracted in December 2020, March 2022, and December 2022 for the respective three batches. LTL assays were performed within a week of DNA extraction. Twenty-four DBS samples from the first batch were re-extracted during the second batch, and 14 samples from the second batch were re-extracted during the third batch to adjust for batch differences. The details of telomere length method have been described previously (Cawthon, 2002; Lin et al., 2010) and can be found on the Telomere Research Network’s website (https://trn.tulane.edu/wp-content/uploads/sites/445/2021/07/Lin-qPCR-protocol-01072020.pdf). The PCR efficiencies of the T and S reactions are 88.4 ± 4.1% and 91.4 ± 3.9%, respectively. The average inter-assay coefficient of variation (CV) for the samples in this study was 2.0 ± 1.5%. Intra-class correlation (ICC) of repeat extractions of 46 dried blood spot samples from this study was 0.883 (95% CI 0.766–0.942). LTL was calculated using T/S ratio (telomere signal to single-copy gene signal) units as a continuous variable.

### Outcomes of Interest

The primary outcomes of interest were the associations between moderate to late preterm birth and postnatal maternal LTL as a continuous variable.

Preterm birth was defined as delivery before 37 completed weeks of gestation (WHO, 2023a), following the World Health Organization (WHO) classification with our study focusing on moderate to late preterm births (32–<37 weeks) (WHO, 2023b). Participants were categorized into two groups: those with and without preterm birth.

### Statistical Analysis

Descriptive analyses were performed for the baseline characteristics of the study population. Continuous variables were presented as either means and standard deviations (SD) or median and interquartile range (IQR), according to data distribution. Categorical variables were presented as frequencies and percentages.

Bivariate analyses were performed to assess the association between maternal LTL (continuous) and maternal covariates for mothers with and without preterm infants. The Welch two sample t-test was used to compare the mean of continuous variables between the two groups. Pearson’s chi-squared test and Fisher’s exact test (for smaller cell sizes) were employed to examine the distribution of categorical variables between the two groups.

#### Confounders

To explore the associations between preterm birth and maternal LTL, we used logistic regression models. Confounders were identified a priori based on prior literature showing associations with both preterm birth risk and maternal LTL, including maternal age, ethnicity, education level, marital status, 100% juice consumption, delivery type, and birthweight (Englund-Ögge et al., 2012; Granés et al., 2023; Leung et al., 2014; Mitchell et al., 2018; Panelli et al., 2022b; Wojcicki et al., 2016).

In addition to confounders identified a priori, we ran unadjusted bivariate logistic regression models to test other potential covariates computing an estimate of the odds ratio (OR) and its associated confidence interval (CI) and p-values. Covariates were included as potential confounders if they showed an association with preterm birth with a p-value < 0.20 in bivariate logistic regression analysis *and* had been shown to be associated with LTL in previous studies. Variables assessed included maternal health, demographics and infant specifics. In general, we attempted to use continuous variables when possible. We chose birthweight as a continuous variable because it allows for a more nuanced capture of fetal growth variations. This approach improves model precision by accounting for subtle differences in weight, better estimating its impact on preterm birth risk and LTL, independent of gestational age, rather than limiting the analysis to categorical classifications like weight-for-age categories. While Cesarean section and birthweight are not predictive of preterm birth given the timing of these relationships, other unmeasured biomarkers associated with preterm birth may be associated with Cesarean section and birthweight.

#### Multivariable Models

The final multivariable logistic model for preterm birth included LTL (T/S ratio) (continuous), maternal age (continuous variable), parity, maternal high school diploma (yes/no), maternal pre-pregnancy obesity (body mass index in kg/m^2^), delivery type (Cesarean section versus vaginal), and infant birth weight (kg, continuous variable). Covariates with less than 5 counts per cell such as hypertension, diabetes, and smoking and/or secondhand smoke exposure were excluded from any multivariable modeling. The model was fit using the variables specified above, without any automatic stepwise predictor selection. Variance inflation factor (VIF) was used to assess multicollinearity between predictor variables in the adjusted logistic regression model. The linearity assumption for the logistic regression model was assessed by examining a scatter plot of the continuous predictor variable against the log-odds of preterm birth. The Hosmer–Lemeshow goodness-of-fit test was performed. A p-value > 0.05 from the test indicates that the model provides a good fit to the data.

#### Additional Multivariable Models

To explore the potential effect of birthweight (continuous variable) on any predictors, a model without the adjustment for birthweight was also constructed. Additionally, a model incorporating interaction terms for LTL and maternal age, both continuous and categorized (< 35 vs. ≥ 35 years) was computed to explore potential statistical interactions. Additionally, age-stratified logistic regression analysis was conducted to explore potential age-specific associations between maternal LTL and the risk of preterm birth.

Finally, we conducted a linear regression with LTL as the outcome to examine factors influencing LTL. This model assessed the association between preterm birth and LTL, adjusting for the same covariates described above that were associated with LTL and preterm birth. Assumptions were checked using visual inspection for linearity, the Breusch-Pagan test for homoscedasticity, and the Shapiro–Wilk test for normality. Due to minor residual deviations, we used robust standard errors, 95% confidence intervals, and p-values to ensure accurate results. All statistical analyses were performed using R Studio (Version 2023.06.0 + 421) and Stata 15.0. The significance level was set at p < 0.05 for all final results.

#### Missing Data

The median missing rate (IQR) was 0 (0.52). The final logistic regression model had 188 variables versus 194 or 3.5% missing. Missing data in continuous variables were addressed using median imputation, given their non-normal distribution. Categorical variables with missing values were addressed using mode imputation. These approaches were selected due to the small proportion of missing data and the assumption that the missingness was missing completely at random (MCAR), consistent with the relatively low observed missing rate.

## Results

### Overall Demographics of the Study Population

A total of 194 participants were included. Among all participants, 23 (11.9%) had preterm infants and 171 (88.1%) had term infants (Table 1). The median maternal age at delivery was 33 years (IQR 28–36). Forty-two percent of participants were classified as having advanced maternal age (> 35 years), while only 3.1% experienced teenage pregnancy (< 18 years). The majority (90%) were married or living with partner (Table 1). The study sample had 73% of mothers with a high school diploma or more education. Seventy-nine percent of the mothers identified as Latina, based on self-report and 65% of fathers with 82% of infants having one parent or more with Latino ethnicity (Table 1). Among the mothers that self-identified as Latina, 36.7% cited Mexican origin and 54.2% cited Central American with the remainder having Caribbean, South American or Spanish ancestry (results not shown).

**Table 1:** Baseline Characteristics of Mothers and Infants in Relation to Preterm Delivery

	Overall, N = 194^1^	Preterm		p-value^2^
		Yes, N = 23 Median (IQR); N, %	No, N = 171 Median (IQR); N, %	
Maternal sociodemographic factors				
Maternal age (years)	33 (28, 36)	31 (27, 35)	33 (28, 37)	0.381
Advanced maternal age (> 35 years)	81 (42%)	6 (26%)	75 (44%)	0.105
Marital status				0.252
Single	19 (9.8%)	4 (17%)	15 (8.8%)	
Married or living with partner	175 (90%)	19 (83%)	156 (91%)	
High school diploma (yes)	141 (73%)	15 (65%)	126 (74%)	0.392
Education level				0.224
High school or lower	95 (49%)	14 (61%)	81 (47%)	
Any college or higher	99 (52%)	9 (39%)	90 (53%)	
Latinx family ethnicity characteristics (based on self-report)				
Latinx child, n (%)	159 (82%)	19 (83%)	140 (82%)	0.999
Latina mother, n (%)	153 (79%)	19 (83%)	134 (78%)	0.789
Latino father, n (%)	127 (65%)	17 (74%)	110 (64%)	0.364
Maternal pre-pregnancy and gestational health characteristics				
Maternal LTL at delivery (T/S) (IQR)	1.43 (1.21, 1.59)	1.52 (1.35, 1.73)	1.42 (1.20, 1.57)	0.045
Age of menarche, years	13.0 (12.0, 14.0)	13.0 (12.0, 13.0)	13.0 (12.0, 14.0)	0.980
Parity	2.0 (1.0, 3.0)	2.0 (1.0, 3.0)	2.0 (1.0, 3.0)	0.914
Maternal weight gain psychological, and metabolic health				
Pre-pregnancy BMI (kg/m^2^)	27 (24, 32)	29 (24, 33)	27 (24, 31)	0.859
Maternal pre-pregnancy obesity	61 (31%)	10 (43%)	51 (30%)	0.185
Pregnancy weight gain (kg)	12.7 (9.1, 15.8)	11.3 (7.9, 14.1)	13.0 (9.1, 15.9)	0.283
Hypertension in pregnancy (pre-existing or gestational)	35 (18%)	6 (26%)	29 (17%)	0.384
Diabetes mellitus (pre-existing or gestational)	34 (18%)	3 (13%)	31 (18%)	0.771
Depression or anxiety (pre-existing or diagnosed in pregnancy)	22 (11.4%)	1 (4.6%)	22 (12.9%)	0.257
Environmental and diet exposure in pregnancy				
Smoking and/or secondhand smoke exposure	13 (6.7%)	3 (13%)	10 (5.8%)	0.188
Sugar sweetened beverage (SSB) consumption in pregnancy	116 (60%)	15 (65%)	101 (59%)	0.572
SSB category, cups/week				0.529
≤ 1 cup/week	113 (58%)	12 (52%)	101 (59%)	
> 1 cup/week	81 (42%)	11 (48%)	70 (41%)	
100% fruit juice consumption in pregnancy, cups/week	110 (57%)	14 (61%)	96 (56%)	0.667
100% fruit juice, cups/week				0.105
≤ 1 cup/week	113 (58%)	17 (74%)	96 (56%)	
> 1 cup/week	81 (42%)	6 (26%)	75 (44%)	
Infant birth characteristics				
Child’s sex				0.285
Female	79 (41%)	7 (30%)	72 (42%)	
Delivery type				< 0.01
Vaginal	142 (74%)	11 (48%)	131 (77%)	
Cesarean section	51 (26%)	12 (52%)	39 (23%)	
Gestational age (weeks), mean (SD)	38.7 (1.8)	35.3 (1.6)	39.2 (1.2)	< 0.001
Birthweight (kg), mean (SD)	3.33 (0.54)	2.78 (0.65)	3.41 (0.48)	< 0.001
Infant weight-for-age classification				0.104
Appropriate for gestational age (AGA)	145 (75%)	14 (61%)	135 (79%)	
Large for gestational age (LGA)	30 (15%)	6 (26%)	24 (14%)	
Small for gestational age (SGA)	15 (7.7%)	3 (7.7%)	12 (7.0%)	
Low birthweight (< 2500 g)	12 (6.2%)	7 (30%)	5 (2.9%)	< 0.001
Birth length (cm)	50.0 (48.3, 52.0)	47.5 (45.5, 49.0)	50.8 (48.5, 52.1)	0.068
Head circumference (cm)	34.2 (33.0, 35.0)	33.0 (31.6, 34.0)	34.5 (33.0, 35.5)	< 0.01
Apgar scores at 5 min	9.0 (9.0, 9.0)	9.0 (9.0, 9.0)	9.0 (8.0, 9.0)	0.277

The median maternal LTL was 1.43 T/S (IQR 1.21–1.59). Almost one-third of mothers (31%) had maternal pre-pregnancy obesity. Hypertension and diabetes were observed in 18% of the participants and 11.4% were diagnosed with or had pre-existing depression and/or anxiety during pregnancy. Thirteen of the participants (6.7%) had secondhand smoke exposure, and among those 13, only 1 individual was also identified as a smoker. The mean gestational age for the infants was 38.7 ± 1.8 weeks and the mean birthweight was 3.33 ± 0.54 kg.

### Baseline Characteristics of Mothers With and Without Preterm Infants

The demographics and baseline characteristics of mothers with and without preterm infants are presented in Table 1.

**Fig. 1:**
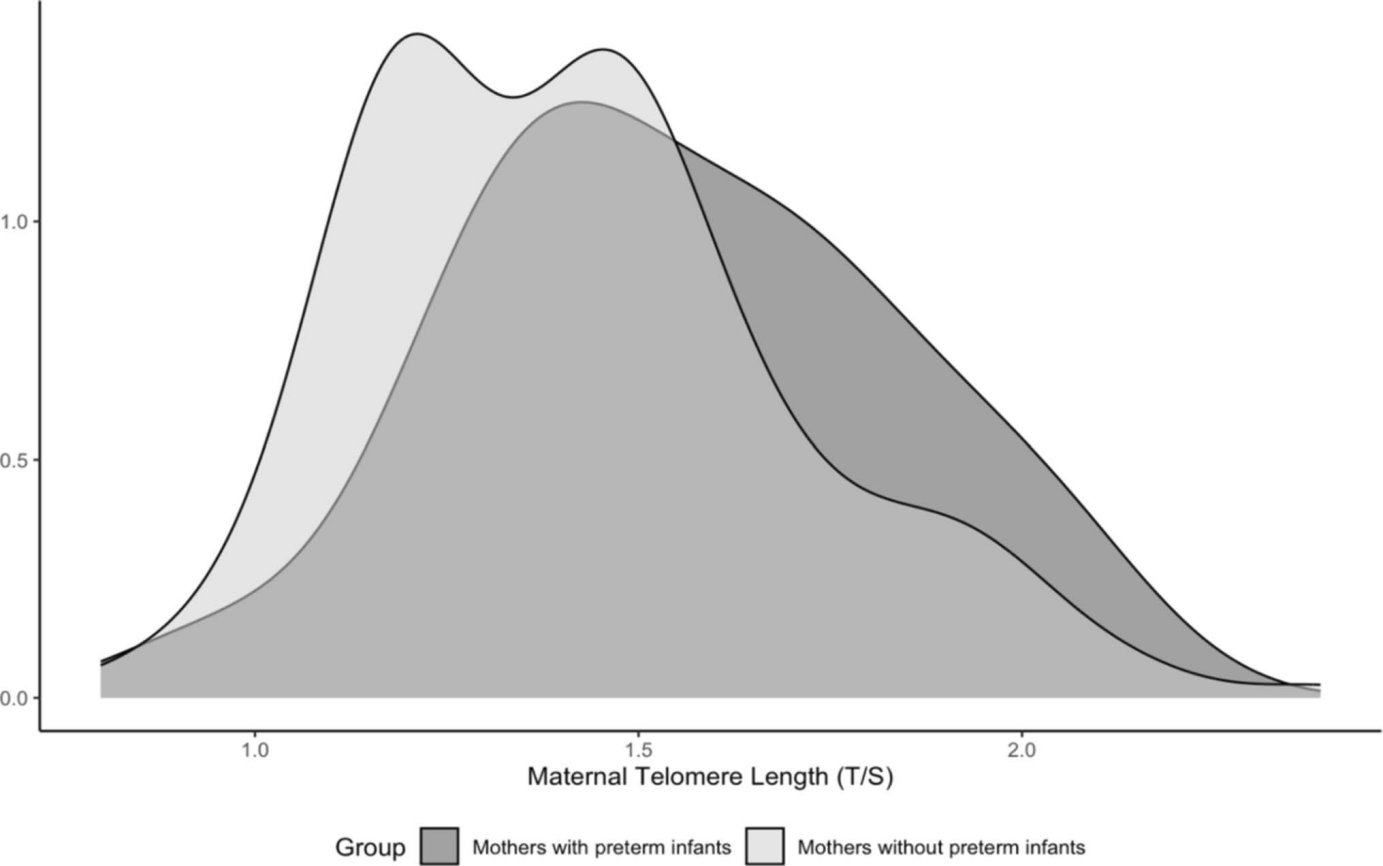
Proportional distribution of maternal LTL (T/S) in relation to preterm birth. The telomere length among mothers with preterm infants (median 1.52, IQR 1.35–1.73) is longer compared to mothers without preterm infants (median 1.42, IQR 1.20–1.57, p = 0.045)

Mothers with preterm birth had a longer median maternal LTL (1.52 T/S vs. 1.42 T/S, p = 0.045). The proportional distributions of LTL between the two groups are shown in Fig. 1. No significant differences were found in other baseline maternal sociodemographics and health characteristics between groups including maternal age, pre-pregnancy BMI, diabetes, hypertension, depression, smoking, marital status, education levels, and sugar sweetened beverage (SSB) and juice consumptions. Mothers with preterm infants had a higher percentage of cesarean sections compared to those without preterm infants (52% vs. 23%, p < 0.01). The mean ± SD gestational ages for the preterm and term infants were 35.3 ± 1.6 and 39.2 ± 1.2 weeks respectively. Birth weight was lower in preterm infants compared to full-term infants (mean 2.78 vs. 3.40 kg, p < 0.001) and preterm infants were also more likely to be SGA (52% vs. 7%; p < 0.001; Table 1).

### Univariate and Multivariable Logistic and Linear Regression Models

The univariate logistic regression analysis for preterm birth is presented in Table 2. Maternal LTL was significantly associated with preterm birth with an OR of 4.68 (95% CI 1.07–20.6, p = 0.039). Low birthweight, small for gestational age, and Cesarean section were also significantly associated with preterm birth (OR = 14.5, 95% CI 4.18, 54.3, OR = 13.5, 95% CI 4.91,38.9, and OR = 3.69, 95% CI 1.51, 9.015, respectively). Other variables did not show statistically significant associations with preterm birth (Table 2).

**Table 2:** Univariate Logistic Regression Analysis of Preterm Birth

	OR	95% CI	p-Value
Maternal sociodemographic factors			
Maternal age (years)	0.96	0.90, 1.04	0.322
Advanced maternal age (> 35 years)	0.45	0.16, 1.15	0.111
Latina (self-identified)	1.31	0.46, 4.73	0.640
Marital status—Married or living with partner	0.46	0.15, 1.73	0.201
High school diploma	0.67	0.27, 1.76	0.395
Education levels—College or higher	0.58	0.23, 1.39	0.228
Maternal pre-pregnancy and prenatal health factors			
Maternal LTL (T/S units)	4.68	1.07, 20.6	0.039
Parity	1.03	0.65, 1.58	0.898
Age of menarche (years)	1.00	0.76, 1.30	0.979
Maternal weight gain, psychological and metabolic health			
Pre-pregnancy BMI (kg/m^2^)	1.00	0.94, 1.06	0.883
Maternal pre-pregnancy obesity	1.81	0.73, 4.38	0.190
Pregnancy weight gain, kg	0.97	0.89, 1.03	0.378
Hypertension in pregnancy (pre-existing or gestational)	1.73	0.58, 4.57	0.290
Diabetes mellitus in pregnancy or pre-existing	0.68	0.15, 2.14	0.549
Depression/anxiety in pregnancy or pre-existing	0.32	0.04, 2.52	0.281
Environmental and diet exposure in pregnancy			
Smoking and/or secondhand smoke exposure	2.41	0.51, 8.70	0.208
Sugar sweetened beverage (SSB) consumption, cups/week	1.30	0.53, 3.38	0.573
SSB > 1 cups/week	1.32	0.54, 3.18	0.530
100% Fruit juice consumption in pregnancy, cups/week	1.22	0.51, 3.06	0.668
100% Fruit juice > 1 cups/week	0.45	0.16, 1.15	0.111
Infant birth characteristics			
Child’s sex—Male	1.66	0.67, 4.52	0.289
Delivery type—Cesarean section	3.69	1.51, 9.15	0.004
Birthweight (kg)	0.10	0.03, 0.25	< 0.001
Small for gestational age (SGA) (reference: appropriate for gestational age (AGA))	2.41	0.51, 8.75	0.211
Large for gestational age (LGA) (reference: (AGA)	2.41	0.79,6.67	0.101
Low birthweight (< 2500 g)	14.5	4.18, 54.3	< 0.001
Birth length (cm)	0.82	0.70, 0.93	0.007
Head circumference (cm)	0.82	0.64, 0.98	0.057
Apgar scores at 1 min	0.92	0.71, 1.27	0.581
Apgar scores at 5 min	0.69	0.43, 1.21	0.159

*OR* odds ratio, *CI* confidence interval, *BMI* body mass index, *N* number of participants, *n* number of participants, *SD* standard deviations, *SSB* sugar sweetened beverage, *T/S* telomere/single copy gene

In a multivariable logistic model for preterm birth including the following nine variables: maternal LTL, maternal age (years), Latina mother (yes/no), maternal pre-pregnancy obesity (yes versus no), parity, married or living with partner (versus single), maternal high school diploma (yes versus no), 100% fruit juice (> 1 cup/week versus less), birhtweight (kg) and delivery type (Cesarean section versus vaginal birth), longer maternal LTL continued to show a significant association with preterm birth (OR 12.8, 95% CI 1.83, 99.9; p = 0.010; Table 3). Cesarean section was also associated with preterm birth (OR 4.30, 95% CI 1.25, 15.6, p = 0.021; Table 3). When we removed Cesarean section from the model, the effect size of the association between LTL and preterm birth decreased slightly (OR 11.1, 95% CI 1.73, 78.5, p = 0.011; results not shown).

**Table 3:** Multivariable Logistic Regression Analysis for Preterm Birth (n = 194)

Multivariable Logistic Regression Model for Preterm Birth	OR	SE	95% CI	p-Value	VIF
Maternal LTL (T/S)	12.8	1.01	1.83, 99.9	0.010	1.3
Maternal age (years)	0.97	0.054	0.87, 1.08	0.588	1.7
Latina mother (versus not)	0.75	0.808	0.15, 3.91	0.722	1.5
Maternal pre-pregnancy obesity (yes versus overweight or normal)	1.76	0.615	0.51, 5.92	0.361	1.2
Parity	1.10	0.290	0.62, 1.96	0.738	1.4
Married or living with partner (versus single)	0.45	0.777	0.10, 2.23	0.45	1.2
Maternal high school diploma (yes versus no diploma)	0.46	0.720	0.11, 1.89	0.277	1.6
100% Fruit juice > 1 cups/week (versus less)	0.20	0.713	0.04, 0.74	0.015	1.3
Delivery type—Cesarean section (versus vaginal)	4.30	0.635	1.25, 15.6	0.021	1.3
Infant birthweight (kg)	0.10	0.580	0.03, 0.28	< 0.001	1.1

Null deviance = 141; Null degree of freedom (df) = 193; Log-likelihood = − 46.9; Akaike Information Criterion (AIC) = 116; Bayesian Information Criterion (BIC) = 152; Deviance = 93.8; Residual df = 183; The Hosmer–Lemeshow goodness-of-fit test: Statistic = 13.1, df = 9, p-value = 0.157

*OR* odds ratio, *SE* standard error, *CI* confidence interval, *VIF* variance inflation factor, *T/S* telomere/single copy gene

We also examined the model without the adjustment for infant birthweight (kg). The effect size for maternal LTL from the model without birthweight was lower than the OR from the model with birthweight (OR = 8.97; 95% CI 1.62, 53.8; p = 0.012; results not shown). There was no significant interaction between maternal LTL and maternal age (both categorized and continuous). The interaction term for age as categorical variable and LTL was OR 0.43 (95% CI 0.01, 27.8, p = 0.688) and age as a continuous variable with LTL was OR 0.84 (95% CI 0.61, 1.15; p = 0.280; results not shown). When stratified by 35 years of age, maternal LTL was significantly associated with an increased odds of preterm birth in the age group under 35 years in an adjusted model with a larger effect size than the pooled OR (OR of 32.5, 95% CI 2.58, 597.0, and p < 0.01 for age < 35; Table 1). There was no association among those 35 and over (Table 4). Preterm birth was positively associated with maternal LTL (as the outcome) in a multivariable model with the same variables as the logistic model(β = 0.17, p = 0.016) (results not shown). Maternal age was negatively associated with LTL (β = –0.01, p = 0.002). High school education is positively associated with LTL (β = 0.14, p = 0.008). Other factors did not show significant associations. The model explained 13.9% of the variance in LTL (R^2^ = 0.139).

**Table 4:** Logistic Regression Analysis of Preterm Birth Stratified by Maternal Age Group: < 35 and ≥ 35 years

Age-Stratified Logistic Regression Analysis of Preterm Birth	Maternal age < 35 years old n = 113			Maternal age ≥ 35 years old n = 81			
	OR	95% CI	p-Value		OR	95% CI	p-Value
Maternal LTL (T/S)	32.5	2.58, 597.0	< 0.01		70.1	0.31, 203,055	0.133
Latina (mother) versus not	0.15	0.01, 1.73	0.122		6.17	0.36, 378	0.222
Maternal pre-pregnancy obesity (vs. overweight/normal)	1.01	0.20, 4.68	0.988		2.39	0.04, 81.1	0.619
Parity	1.25	0.64, 2.55	0.514		1.36	0.37, 6.01	0.635
100% Juice consumption > 1 cups/week (vs. less)	0.18	0.03, 0.93	0.020		0.30	0.01, 4.62	0.417
Married or living with partner (vs. single)	0.38	0.07, 2.01	0.249		41,585	0.00, NA	0.983
High school diploma (vs. no high school)	0.18	0.03, 0.77	0.041		8.0e + 7	0.00, NA	0.260
Cesarean section (vs. vaginal)	6.73	1.40, 40.2	0.017		12.6	0.31, 1,982	0.184
Birthweight (kg)	0.19	0.05, 0.57	0.007		0.00	0.00, 0.09	< 0.001

*OR* odds ratio, *CI* confidence interval, *T/S* telomere/single copy gene

## Discussion

In this study, we investigated the association between maternal LTL in the immediate postpartum period and preterm birth in a cohort of primarily Latina mothers. Our findings suggest an association between longer maternal LTL and moderate-late preterm birth in a cohort of infants that excluded very preterm infants (< 32 weeks) and those with significant co-morbidities. After adjusting for potential confounders, the effect size increased suggesting an independent association between longer maternal LTL in the postpartum period and preterm birth compared with term births. The association between maternal LTL and preterm birth was most significant in mothers primarily below 35 years of age.

Our results contrast with previous studies which found shorter maternal LTL associated with preterm birth (Page et al., 2021; Panelli et al., 2022a). The only other study to our knowledge that focused on LTL in pregnant Latina women was the study by Page et al. (2021), which focused on Mexican origin women with a much smaller sample size. Their preterm group consisted of only 5 out of a total sample size of 100, with blood samples collected for telomere analysis between 26 and 36 weeks gestation (Page et al., 2021). These previous studies all collected LTL during the prenatal period in contrast with our focus on LTL collected in the immediate postnatal period at the time of delivery.

Discrepancies in the timing of blood sample collection for LTL and potential postpartum factors could contribute to the observed variations in LTL findings (Panelli et al., 2022a). The previous studies collected their LTL samples during prenatal visits (Page et al., 2021; Panelli et al., 2022a), while we collected our LTL samples in the immediate postpartum period. Studies by Panelli et al. (2022a) and Mitchell et al. (2018) found stable maternal LTLs throughout pregnancy and the postpartum period, but postpartum LTLs were significantly shorter in individuals who had Cesarean delivery compared to vaginal delivery (Panelli et al., 2022a). Despite the higher proportion of Cesarean sections in the preterm group (52% vs. 23%, p = 0.003) in our study, we evaluated the association removing any adjustment for Cesarean section finding that preterm birth remained significant regardless of the presence of Cesarean sections in the model. It is not clear why LTL would be longer in our sample population of women with preterm deliveries although it is possible that there are physiologic differences between LTL in the postnatal versus prenatal setting.

Additionally, demographic composition could contribute to the inconsistency between their findings and ours. The study by Page et al. (2021) specifically focused on individuals of Mexican origin, while the study by Panelli et al. (2022a) had a diverse composition with almost 50% being White, followed by Asians and Pacific Islanders. In contrast, our study comprised a more heterogeneous overall sample with 79% Latina and heterogeneity within this Hispanic population.

Previous research has shown that shorter telomeres are associated with depression and anxiety (Malouff & Schutte, 2017; Ridout et al., 2016). The lower prevalence of depression and anxiety in our study cohort (11.4%) may also account for observed differences in LTL related to preterm birth. In larger studies with similar low prevalence rates of maternal stress, depression, and/or anxiety (ranging from 6 to 11%), Sand et al. (n = 318) and Ämmälä et al. (n = 1405) found no association with maternal LTL (Ämmälä et al., 2020; Send et al., 2017). In contrast, a study of 109 pregnant women by Garcia-Martin et al. with a higher proportion of women with depression and/or anxiety (27.5%) found an association between prenatal depression and shorter placental telomeres (Garcia-Martin et al., 2021). Similarly, Panelli et al. reported a higher prevalence of depression and/or anxiety (20.5%) among their 44 pregnant participants compared to our study (Panelli et al., 2022a).

Furthermore, the discrepancy between our findings and previous studies may be due to our cohort consisting of moderate-to-late preterm births with minimal co-morbidities, which could be influenced by the Hispanic paradox, where favorable birth outcomes are observed in Hispanic women despite potential barriers related to socioeconomic status and healthcare access (Montoya-Williams et al., 2021). Factors such as strong social support, cultural practices, and protective health behaviors may contribute to this paradox (Montoya-Williams et al., 2021). However, this phenomenon is not fully understood, and factors like immigration status and length of U.S. residence could affect our findings.

The observed longer LTL in mothers with preterm infants in our study may indicate unique pathophysiological pathways, possibly contributing to disease development. Longer telomeres have been found to be associated with specific chronic diseases including hypertension, multiple sclerosis, specific cancers and systemic lupus erythematosus in certain populations (Chen et al., 2023; Wang et al., 2022). However, the exact mechanisms behind these associations remain unclear. Some studies found a U-shaped, non-linear relationship between LTL and risk for pancreatic, colorectal, and breast cancers (Cui et al., 2012; Qu et al., 2013; Skinner et al., 2012) emphasizing that optimal LTL equilibrium is vital for physiological functionality; deviations, either excessive shortening or elongation, may precipitate pathological development. Similarly, this U-shaped pattern may extend to preterm birth, where both excessively short and long LTL could be associated with risk. We also found that the association between longer LTL and preterm birth was strongest among women <35 years possibly suggestive of a different mechanistic pathway than preterm births associated with younger or older maternal age.

In addition to or in conjunction with LTL, other maternal biomarkers are used to assess physiological risk for preterm birth including the neutrophil-to-lymphocyte ratio (NLR), matrix metalloproteinases (MMP-8, MMP-9), and microRNAs/DNA methylation (Liu et al., 2017; Toure et al., 2018; Vakili et al., 2021). NLR reflects inflammatory balance, while MMPs are linked to cervical remodeling, and microRNAs/DNA methylation capture dynamic gene regulation in response to stress and inflammation (Liu et al., 2017; Toure et al., 2018; Vakili et al., 2021). Unlike these other markers, however, which provide more immediate or pathway-specific insights, LTL offers a unique measure of chronic stress and long-term cellular aging, making it valuable for understanding the cumulative biological effects over gestation that may contribute to preterm birth. Future comparative studies could evaluate LTL in relation to these other markers at different time points in the perinatal period and preterm birth outcomes.

### Limitations

This study has several limitations. The study design involves cross-sectional data collection, as LTL was measured at recruitment, shortly after delivery. In cross-sectional studies, exposure and outcome are assessed simultaneously, so causality cannot be established. We also lacked sufficient information to distinguish between spontaneous and medically indicated preterm birth. Although we can adjust for Cesarean section delivery, other key data needed to differentiate the two, such as labor induction, maternal or fetal conditions prompting preterm delivery (including preeclampsia, placental abruption, intrauterine growth restriction), were not available in our dataset. We also lack data on immigration status and length of U.S. residence, factors linked to protective effects against adverse birth outcomes particularly among Latina women (Sinclair et al., 2020) that could affect stress-related pathways and LTL dynamics.

The multifactorial nature and complexity of preterm birth complicate efforts to generate accurate prediction models, as factors such as inflammation, infection, and genetics may vary in their impact. Future studies should assess the change in LTL during the perinatal period (from prenatal to postnatal) to better characterize how stress, inflammation and other exposures can impact risk for preterm birth. Additionally, we excluded early preterm infants and those with significant morbidities and as such our study cannot be generalizable to all preterm infants; future studies should include all preterm births to better understand the relationship between LTL and physiological pathways.

Although we have a modest sample size, a larger sample size is needed for a more precise and accurate results for our outcome of interest, preterm birth which was present in slightly more than 10% of our cohort. Additionally, for the age-stratified analysis, the older age group (≥ 35 years) had a smaller sample size, and a larger sample size in that group may have yielded different results.

## Conclusions

Our study examined the association between maternal LTL and moderate-to-late preterm birth among Latina mothers. The results of our study showed that mothers with moderate-to-late preterm infants had longer LTL in the immediate postnatal period compared to those with term infants. This association was particularly notable among younger mothers, specifically those under the age of 35. The significant association observed between maternal LTL and preterm birth highlights the potential significance of LTL as a biomarker within the Hispanic/Latinx population. Further research is warranted to gain a better understanding of the underlying mechanisms involved and to explore potential clinical implications associated with maternal LTL in the context of preterm birth.

## Data Availability

The datasets used and/or analyzed during the current study are available from the corresponding author on reasonable request.

## Abbreviations

BMI: Body mass index
CI: Confidence interval
CV: Coefficient of variation
DNA: Deoxyribonucleic acid
DBS: Dried blood spots
hTERT: Human telomerase reverse transcriptase
ICC: Intra-class correlation
IQR: Interquartile range
LTL: Leukocyte telomere length
MCAR: Missing completely at random
MMP: Matrix metalloproteinases
NLR: Neutrophil-to-lymphocyte ratio
OR: Odds ratio
qPCR: Quantitative polymerase chain reaction
RNA: Ribonucleic acid
SD: Standard deviations
SE: Standard error
SSB: Sugar sweetened beverage
T/S: Telomere/single copy gene
VIF: Variance inflation factor
